# Effect modification of the association between fine particulate air pollution during a wildfire event and respiratory health by area-level measures of socio-economic status, race/ethnicity, and smoking prevalence

**DOI:** 10.1088/2752-5309/acc4e1

**Published:** 2023-04-11

**Authors:** C E Reid, E M Considine, G L Watson, D Telesca, G G Pfister, M Jerrett

**Affiliations:** 1 Department of Geography, University of Colorado Boulder, Boulder, CO, United States of America; 2 Department of Applied Math, University of Colorado Boulder, Boulder, CO, United States of America; 3 Department of Biostatistics, Fielding School of Public Health, University of California Los Angeles, Los Angeles, CA, United States of America; 4 National Center for Atmospheric Research, Boulder, CO, United States of America; 5 Department of Environmental Health Sciences, Fielding School of Public Health, University of California Los Angeles, Los Angeles, CA, United States of America; 6 Current address: Department of Biostatistics, Harvard T.H. Chan School of Public Health, Harvard University., Boston, MA, United States of America

**Keywords:** wildfires, respiratory health, effect modification, find particulate matter, smoking, race/ethnicity, socioeconomic status

## Abstract

Fine particulate air pollution (PM_2.5_) is decreasing in most areas of the United States, except for areas most affected by wildfires, where increasing trends in PM_2.5_ can be attributed to wildfire smoke. The frequency and duration of large wildfires and the length of the wildfire season have all increased in recent decades, partially due to climate change, and wildfire risk is projected to increase further in many regions including the western United States. Increasingly, empirical evidence suggests differential health effects from air pollution by class and race; however, few studies have investigated such differential health impacts from air pollution during a wildfire event. We investigated differential risk of respiratory health impacts during the 2008 northern California wildfires by a comprehensive list of socio-economic status (SES), race/ethnicity, and smoking prevalence variables. Regardless of SES level across nine measures of SES, we found significant associations between PM_2.5_ and asthma hospitalizations and emergency department (ED) visits during these wildfires. Differential respiratory health risk was found by SES for ED visits for chronic obstructive pulmonary disease where the highest risks were in ZIP codes with the lowest SES levels. Findings for differential effects by race/ethnicity were less consistent across health outcomes. We found that ZIP codes with higher prevalence of smokers had greater risk of ED visits for asthma and pneumonia. Our study suggests that public health efforts to decrease exposures to high levels of air pollution during wildfires should focus on lower SES communities.

## 1. Introduction

The frequency and duration of large wildfires and the length of the wildfire season have all increased [1] in the western United States, partially due to climate change [2, 3]. Future climate projections indicate wildfire risk, air pollution from wildfires, and associated health risks will all increase [4–6]. Although fine particulate air pollution (PM_2.5_) is decreasing in most areas of the United States, this is not the case in some parts of the western US and this lack of air quality improvement can be attributed to wildfire smoke [7, 8]. Wildfires are known to increase ambient PM_2.5_ [9] and ground-level ozone (O_3_) [10] and exacerbate respiratory health issues [11] with some evidence for increased mortality and cardiovascular health issues [12]. Further understanding of the health impacts of wildfires, especially its effects on vulnerable populations, however, is needed.

Increasingly, evidence suggests differential health impacts of air pollution for different sub-populations, specifically groupings by socioeconomic status (SES) and by race/ethnicity [13]. Relatively few studies have attempted to investigate differential vulnerability by sub-groups with regards to wildfire smoke exposure [11, 12]. Results from these studies are inconsistent [12]. Of the few papers to investigate differential vulnerability to wildfire smoke by SES, those investigating populations in the U.S. found differential results by SES [14–16], whereas a Canadian study found no differential risk [17]. Differences by race/ethnicity in Australia have demonstrated higher impacts among indigenous populations [18, 19]. One U.S. study found higher risks of respiratory hospitalizations during wildfires for elderly Blacks compared to elderly Whites [15], whereas another found higher cardiovascular hospitalizations among non-Hispanic White individuals [20].

SES is a complex metric of social class that incorporates, among other things, income, education, and occupation [21]. Many metrics can be used for each category. Additionally, income, education, and occupation often interact with one another; certain levels and types of education tend to lead to certain occupations and incomes. Evidence also suggests that both individual-level and area-level SES affect health, potentially through different pathways [22]. Because of strong associations between measures of income, education, and occupation and because no one metric of SES fully captures the full construct of social class, many researchers increasingly use composite measures of SES, such as the Socioeconomic Position (SEP) Index [23] and Concentrated Disadvantage [24]. Yet it is unclear which metrics of SES are the most appropriate to use for any given study and thus we chose multiple metrics of the different constructs of SES in an attempt to help elucidate how SES interacts with air pollution to affect health.

Differential health impacts from air pollution in general could be due to differential exposure levels or to differential susceptibility to air pollution or some combination of both [22, 25]. Many studies document higher exposure to air pollution from non-wildfire sources in lower SES areas [21] and communities of color [26, 27]. This could be due to siting of sources of air pollution such as industrial plants or highways [25, 28] in communities with less political power to fight the construction of those sources or due to decreases in property values near air pollution sources leading those with financial means to move away [22]. Recent work to document these environmental injustices has demonstrated that they can be linked to redlining, the act of denying federally-backed home loans to urban neighborhoods considered to be less desirable due to the composition of their neighborhoods with higher proportions of immigrants and persons of color, and other forms of systemic racism [29, 30]. Wildfire smoke exposure, however, may or may not be differential by SES or race/ethnicity. Although studies have found differential impacts by SES or race for vulnerability to wildfire occurrence [31] or area burned [32], smoke exposure is different from area burned or vulnerability to wildfire. Once the wildfire ignites, meteorological conditions can cause air pollution impacts to occur very far from the fire which could have a very different spatial pattern than wildfire risk. Thus, air pollution exposures from wildfires could be differential by SES or race/ethnicity, but not necessarily so. Although Burke *et al* [33] found wildfire PM_2.5_ concentrations to be higher in communities with larger White populations, this was averaged across the US and covered many wildfires over a seven year pattern. The spatial pattern of wildfire PM_2.5_ concentrations for one fire may not follow that pattern. Additionally, differential health impact differs from differential smoke exposure because underlying health conditions and access to health care may also render some populations more susceptible to smoke exposure than others.

Our study aims to investigate if differential associations exist by various area-level measures of SES, racial/ethnic composition, and smoking prevalence between air pollution (both PM_2.5_ and O_3_) exposure during a wildfire and respiratory hospitalizations and emergency department (ED) visits. This work extends upon previous work into differential impacts of wildfire PM_2.5_ by ZIP code-level SES [14] in which it was found that asthma ED visits were significantly associated with PM_2.5_ during the wildfire for all levels of median income, percent owner occupied housing, and percent with less than a high school diploma in the ZIP code, but that some differential impacts of PM_2.5_ on COPD ED visits by ZIP code-levels of median income were present. Here we investigate other metrics of SES including composite measures, metrics of race or ethnicity, and area-level estimates of smoking prevalence. We also investigate for the first time whether health impacts of O_3_ exposure during wildfires on respiratory health stratify by SES or race/ethnicity.

## 2. Methods

### 2.1. Setting

**Figure 1.**
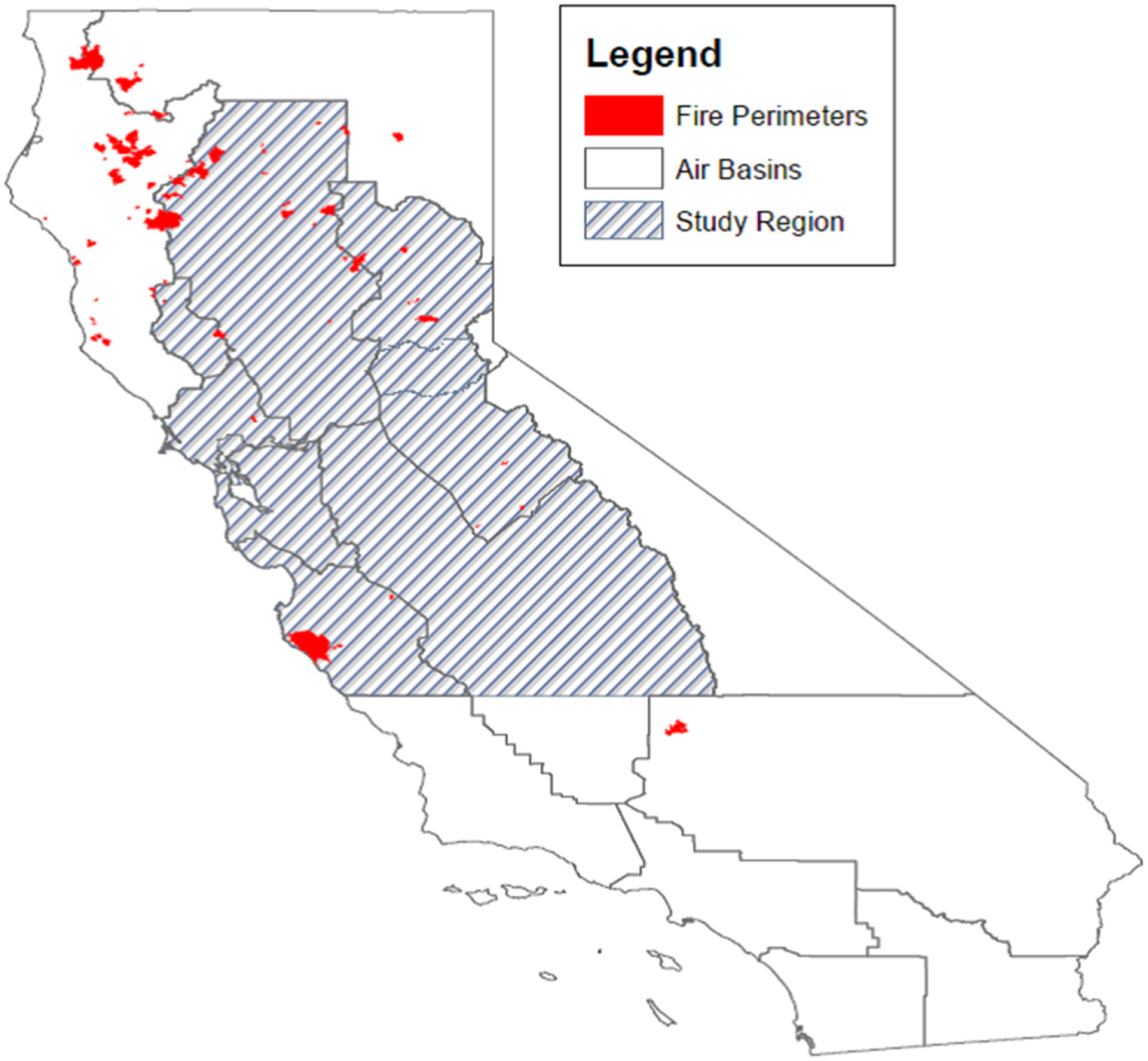
Geographic extent of the study region.

We analyzed data between 20 June and 31 July 2008 from all ZIP codes with more than 100 people (*N* = 751) within the air basins affected by the 2008 northern California wildfires (figure 1). We estimate that over 10 million people living in this region were exposed to elevated levels of both PM_2.5_ and O_3_ during this event. Although this fire occurred a while ago, this fire episode—for its geographic extent, duration, and the large population affected—allows a glimpse into the public health effects of smoke from wildfires. While we await more information about the more recent and devastating wildfire seasons, we can look to the past to assess if there were differential health impacts across a large diverse population affected by this wildfire event.

### 2.2. Data

#### 2.2.1. Outcome data

We obtained daily counts of hospitalizations and ED visits for asthma (ICD-9 code 493), COPD (496 491–492), pneumonia (480–486), acute bronchitis (466), and acute respiratory infections (460–465) by ZIP code in our study area from the California Department of Public Health Environmental Health Investigations Branch.

#### 2.2.2. Exposure data

Because some ZIP codes in our study region do not have air quality monitors, we estimated daily exposures to PM_2.5_ and O_3_ at each ZIP code centroid using estimates of 24 hour PM_2.5_ and 8 hour O_3_ from spatiotemporal machine learning models that have been previously published [34, 35] (supplementary text 1). Briefly, these estimates were created using daily observations from ground monitors for the pollutant of interest as the dependent variable and a variety of environmental covariates such as land use information, daily satellite air pollution measures, meteorological data, and chemical transport model output, as predictor variables. We used leave-one-location-out (LOLO) and ten-fold cross-validation (CV) on ten commonly used machine learning algorithms and found that the generalized boosting model fit both pollutants the best. The ten-fold CV R^2^ were 0.78 and 0.73 and the LOLO CV R^2^ values were 0.60 and 0.68, for PM_2.5_ and O_3_ respectively. The LOLO CV tends to be lower than ten-fold CV but gives a better understanding of performance for spatial prediction [36]. This method calculates total PM_2.5_ rather than PM_2.5_ solely from the wildfire. During wildfires most of the PM_2.5_ is due to wildfire smoke [37] and additionally, the population is breathing all of the air pollution, not just what is directly caused by the fire.

#### 2.2.3. Covariates

We took 24 hour average temperature and relative humidity variables from the Rapid Update Cycle model from the National Climatic Data Center (http://ruc.noaa.gov/) and then used the weathermetrics R package to calculate the heat index with the equation used by the U.S. National Weather Service which is known to be adversely associated with various health outcomes [38] and can be higher during wildfire events.

To adjust for differential population risk for respiratory and cardiovascular diseases at the ZIP code-level, we used ZIP code-level median income, percent of the population over 65, percent of the population under age 5, and percent of the population that was non-White derived from the 2000 U.S. Census. We also adjusted for ZIP-code level smoking prevalence data derived from small-area estimates based on the Behavioral Risk Factor Surveillance System and Census data by ZIP code for the 2006–2010 time period based on the 2000 census ZIP codes [39]. Although smoking prevalence is also considered as an effect modifier, it is controlled for as a confounder in models evaluating other effect modifiers.

#### 2.2.4. Effect modifiers

Due to the complexity of SES as a construct, we used a variety of metrics in an attempt to represent the many domains of SES as effect modifiers of the relationship between wildfire PM_2.5_ and O_3_ and respiratory health. The following data come from the 2000 U.S. Census and are all calculated at the ZIP code level. We used percent of homes worth more than $750 000 as a measure of wealth; unemployment rate and percent working class as measures of occupational class; percent of low income households (median annual income of $20 000 or less) and percent of high income households (median annual income of $ 200 000 or more) as measures of income, and percent living below the poverty line for a measure of poverty. We also calculated the SEP index and Concentrated Disadvantage, both composite measures of SES. These metrics have been used in various studies of air pollution [21] and their derivation are described elsewhere [40]. Details of calculations for all of these metrics are provided in supplementary text 2.

For racial/ethnic composition, we used percent non-Hispanic Black, percent non-Hispanic White, percent non-Hispanic Asian, and percent Hispanic. Percentages of American Indian and Alaska Native, Native Hawaiian and Other Pacific Islander were too small to use in our effect modification analyses.

We additionally interacted each exposure with our ZIP code-level smoking prevalence estimates.

### 2.3. Statistical analyses

First, we investigated if there was differential exposure to PM_2.5_ and O_3_ during the wildfire by assessing the direction of the relationship and statistically significance between the SES, racial/ethnic composition, and smoking prevalence variables and the ZIP-code level pollutant exposures using linear regression.

Then, to quantify the association between each air pollutant and health outcome listed above, we used Poisson generalized estimating equations (GEEs) with an exchangeable correlation structure, with the log-transformed population of each ZIP code as the offset. This statistical model accounts for repeated measures by day at the ZIP-code level. Although space-time generalized linear models often use the mixed models framework, our focus on population-level estimates motivates the use of GEE as a robust alternative. We used the Huber-White sandwich estimator to get standard errors robust to covariance misspecification and overdispersion. The equation used for analyses of PM_2.5_ shown below:

$$Y_{ij}\sim \text{Poisson}\left(\mu_{i,j},\sigma^{2}\right)$$

$$g\left(\mu_{i,j}\right)=\beta_{0}+\text{PM }2.5_{i,j}*\text{effect }{\text{modifier}}_{i}+{\text{ozone}}_{i,j}+\text{heat }{\text{index}}_{i,j}+{\text{weekday}}_{j}+{\text{holiday}}_{j}+\text{ns}\left(\text{julian }{\text{data}}_{j},\text{ df}=3\right)+\text{median }{\text{income}}_{i}+\%\text{over }65_{i}+\%\text{ less than }5_{i}+\%\text{ non}-{\text{white}}_{i}+\text{smoking }{\text{prevalence}}_{i}+\text{offset}\left(\text{log}\left({\text{population}}_{i}\right)\right)$$

where *i* = ZIP code, *j* = day, *g* is the log link, *μ* is the count of the health outcome by day and ZIP code, *β*_0_ is the intercept. When analyzing effect modification for ozone exposure, each effect modifier was interacted (multiplied) with ozone instead of PM_2.5_.

All models included both PM_2.5_ and O_3_, which are not highly correlated in our dataset (*r* = 0.31). To adjust for potential confounding variables, we included estimated smoking prevalence (derived at the ZIP code-level as described above), percent of the ZIP code aged 65 or older, percent of the ZIP code aged 5 or younger, ZIP code-level median income, % of the population that was non-White, daily heat index, temporal trend modeled by a natural cubic spline with 3 degrees of freedom, and dummy variables for weekdays and holidays.

We modeled exposures to both PM_2.5_ and O_3_ as moving averages of the two days prior to the health event. This was based on preliminary analysis of lags up to seven days, which indicated that most of the health impacts for all outcomes analyzed were due to exposures in this timespan. Other studies of the health impacts of wildfire smoke exposure have reported similar findings [17, 41, 42].

Effect modification analyses interacted PM_2.5_ and O_3_ separately with each pre-identified area-level (ZIP code) measure of SES, race/ethnicity, or smoking prevalence, classified into tertiles for each metric for interpretable results (supplementary text 3).

All analyses were done using R version 3.4.4 [43].

## 3. Results

### 3.1. Descriptive statistics

Tables 1 and 2 show the mean and range for health outcomes and effect modifier data used in our analysis, demonstrating more ED visits than hospitalizations.

All of the ZIP code-level effect modifiers were statistically significantly associated with PM_2.5_ and O_3_ concentrations during the 2008 northern California wildfires (table 3). Higher levels of PM_2.5_ occurred in ZIP codes with higher levels of percent low income and working-class residents. We found, however, the opposite for measures of poverty (higher PM_2.5_ in ZIP codes with lower percent poverty), unemployment (higher PM_2.5_ in ZIP codes with lower levels of percent unemployed), and composite measures of SES (higher PM_2.5_ levels in ZIP codes with lower Concentrated Disadvantage and lower levels of the SEP Index (indicating higher SES)). PM_2.5_ values were higher in ZIP codes with higher percentages of White residents and lower percentages of people of color. Associations between O_3_ exposure during the 2008 northern California wildfires and SES and racial/ethnic composition by ZIP code indicated that there was higher exposure to O_3_ in lower SES communities for all SES measures analyzed. This contrasts with some studies that find that of all air pollutants, routine O_3_ concentrations not associated with wildfires tend to be higher in more advantaged areas [44]. For racial and ethnic composition, however, O_3_ concentrations during the fires were higher in ZIP codes with higher proportions of White and Hispanic residents but lower proportions of Black and Asian residents. Both PM_2.5_ and O_3_ levels were higher in ZIP codes with higher smoking prevalence.

### 3.2. Respiratory health and PM_2.5_ exposure during the 2008 California wildfires by SES and race/ethnicity

**Figure 2.**
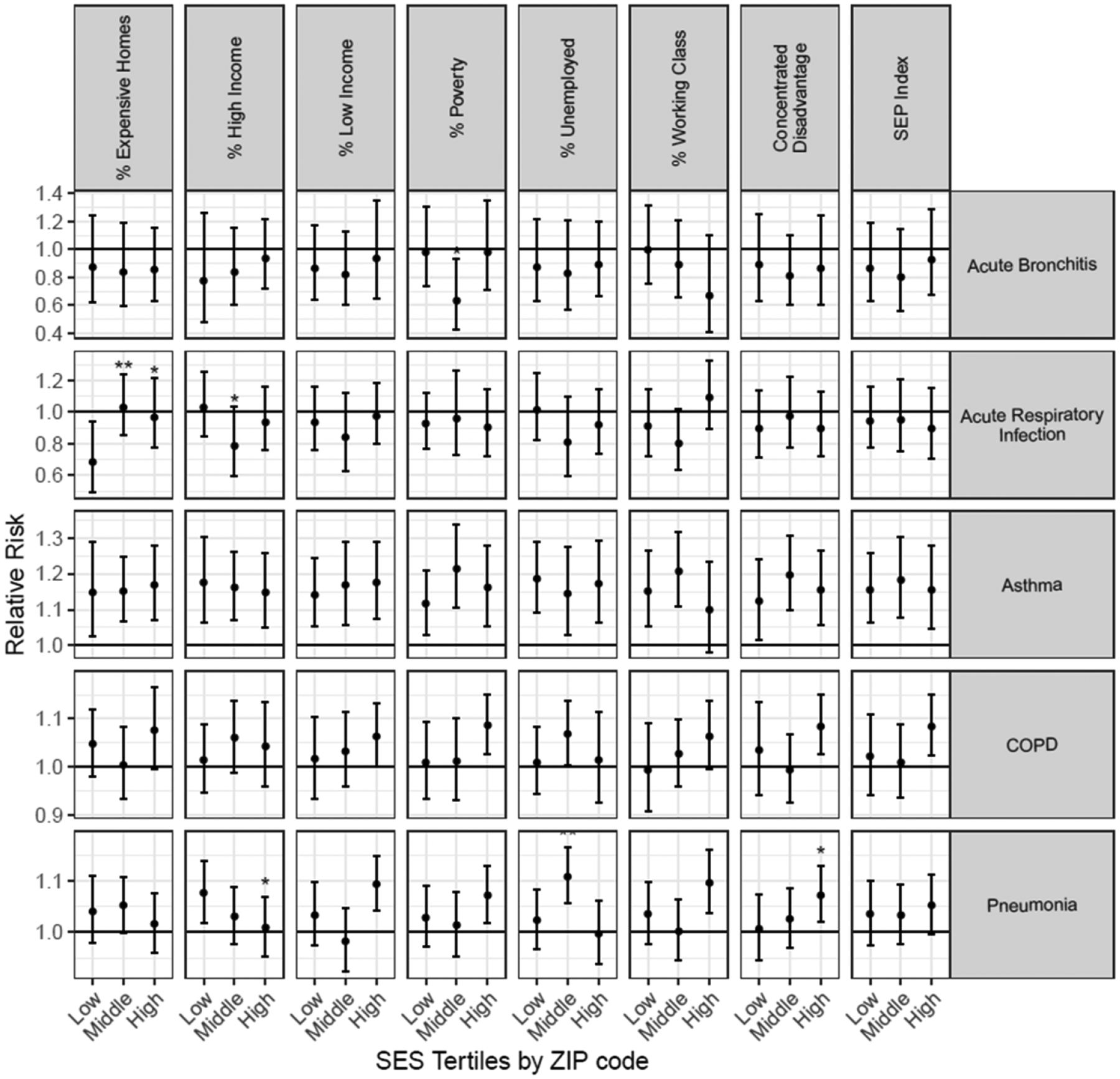
Associations per one *μ*g m^−3^ increase in PM_2.5_ and hospitalizations by levels of ZIP code-level SES during the 2008 northern California wildfires. ** = significantly (*p* < 0.05) different from the lowest tertile; * = significantly (*p* < 0.10) different from the lowest tertile.

Regardless of ZIP code-level SES, asthma hospitalizations were significantly and positively associated with PM_2.5_ during the wildfire (except for the highest tertile of percent working class) in regression models adjusted for confounding variables. COPD hospitalizations were significantly associated with PM_2.5_ mostly in ZIP codes with the lowest SES values as evidenced by the increasing RRs with middle and high tertiles for the SEP Index, Concentrated Disadvantage, and percent living below the poverty line, although none of these interactions were significantly different from each other (figure 2; supplementary table 1). Similarly, pneumonia hospitalizations were significantly associated with PM_2.5_ during the fires most often for the lower SES ZIP codes such as ZIP codes with the lowest levels of high-income homes or the highest levels of Concentrated Disadvantage. This finding was not always consistent because the relationship between PM_2.5_ and hospitalization for pneumonia was significantly higher for the middle tertile of percent unemployed as compared to the lowest and highest tertiles. Results for the association between PM_2.5_ and hospitalizations for acute respiratory infections and acute bronchitis across multiple measures of SES were consistently non-significant.

Increasing tertiles of some SES measures indicate higher SES (e.g. % expensive homes, % high income) whereas higher values of the other SES measures indicate lower SES.

**Figure 3.**
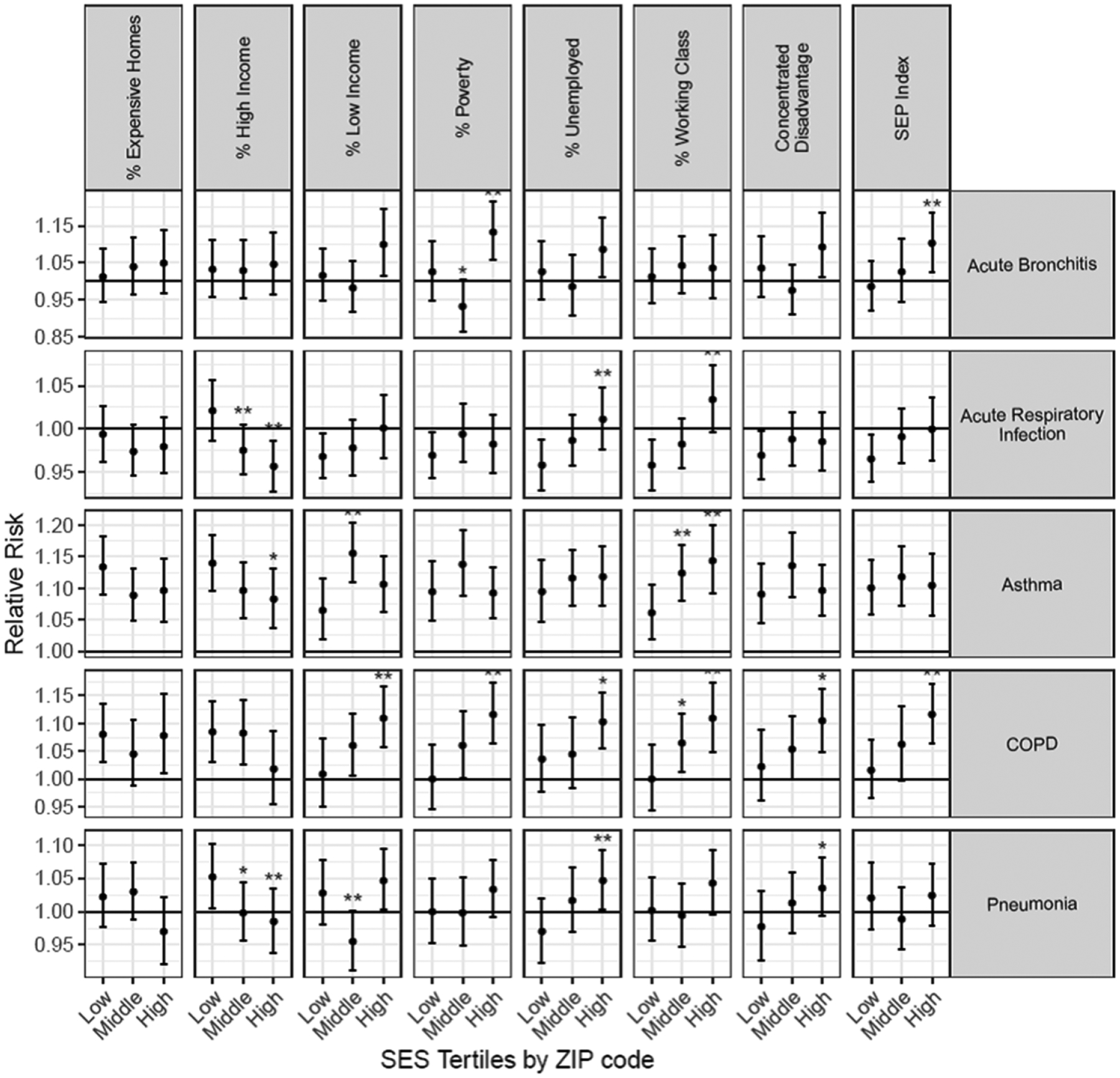
Associations per one *μ*g m^−3^ increase in PM_2.5_ and ED visits by levels of ZIP code-level SES during the 2008 northern California wildfires. ** = significantly (*p* < 0.05) different from the lowest tertile; * = significantly (*p* < 0.10) different from the lowest tertile.

ED visits, like hospitalizations, for asthma were associated with PM_2.5_ at all levels of SES for every metric of SES investigated (figure 3; supplementary table 2). We observed apparent higher risk of COPD ED visits associated with PM_2.5_ with decreasing levels of SES for most SES measures, with the lowest SES tertile being significantly different from the highest SES tertile for most but not all (e.g. % expensive homes and % high income) measures of SES. Associations between PM_2.5_ during the wildfire and ED visits for pneumonia, however, did not exhibit consistently increased risk with decreasing levels of SES, but some evidence suggests increased risk with increasing percent unemployed, increasing Concentrated Disadvantage, and decreasing levels of high-income earners in the ZIP code.

Increasing tertiles of some SES measures indicate higher SES (e.g. % expensive homes, % high income) whereas higher values of the other SES measures indicate lower SES.

We observed some significant findings of effect modification by SES for acute bronchitis ED visits, whereas we did not for acute bronchitis hospitalizations, because of higher counts for ED visits than hospitalizations for this outcome. We found significant associations with PM_2.5_ during these wildfires for acute bronchitis in ZIP codes with the highest levels of poverty, and the SEP Index (in which higher values denote lower SES) compared to the lowest tertile. Although many of the associations for acute respiratory infection ED visits and PM_2.5_ during the wildfire were non-significant, associations were more positive (adverse) with lower SES. This was most visible for percent high income earners, percent working class, and percent unemployed.

Associations between respiratory hospitalizations and PM_2.5_ during the wildfire did not show consistent patterns by ZIP code-level racial composition. We found significant associations for asthma hospitalizations at all levels of percent Hispanic/Latino. Most of the associations for other respiratory hospitalizations were null (supplementary figure 1).

Similar to our results for SES, we found significant associations between PM_2.5_ during the wildfire and asthma ED visits regardless of the racial composition of the ZIP code (supplementary figure 2). Most other results for effect modification by race/ethnicity for respiratory ED visits by PM_2.5_ were null or inconsistent across tertiles.

### 3.3. Respiratory health and ozone exposure during the 2008 California wildfires by SES and race/ethnicity

We did not find consistent effect modification of the association between O_3_ and respiratory hospitalizations by SES measures (supplementary figure 3). Although a few variables showed some evidence of increasing association between O_3_ and respiratory ED visits with lower SES level (supplementary figure 4), these were less consistent across SES variables than they were for PM_2.5_.

Associations between O_3_ and hospitalizations (supplementary figure 5) and ED visits (supplementary figure 6) by ZIP code-level race/ethnicity were largely null.

### 3.4. Respiratory health and PM_2.5_ and ozone exposure during the 2008 California wildfires by ZIP code-level smoking prevalence

**Figure 4.**
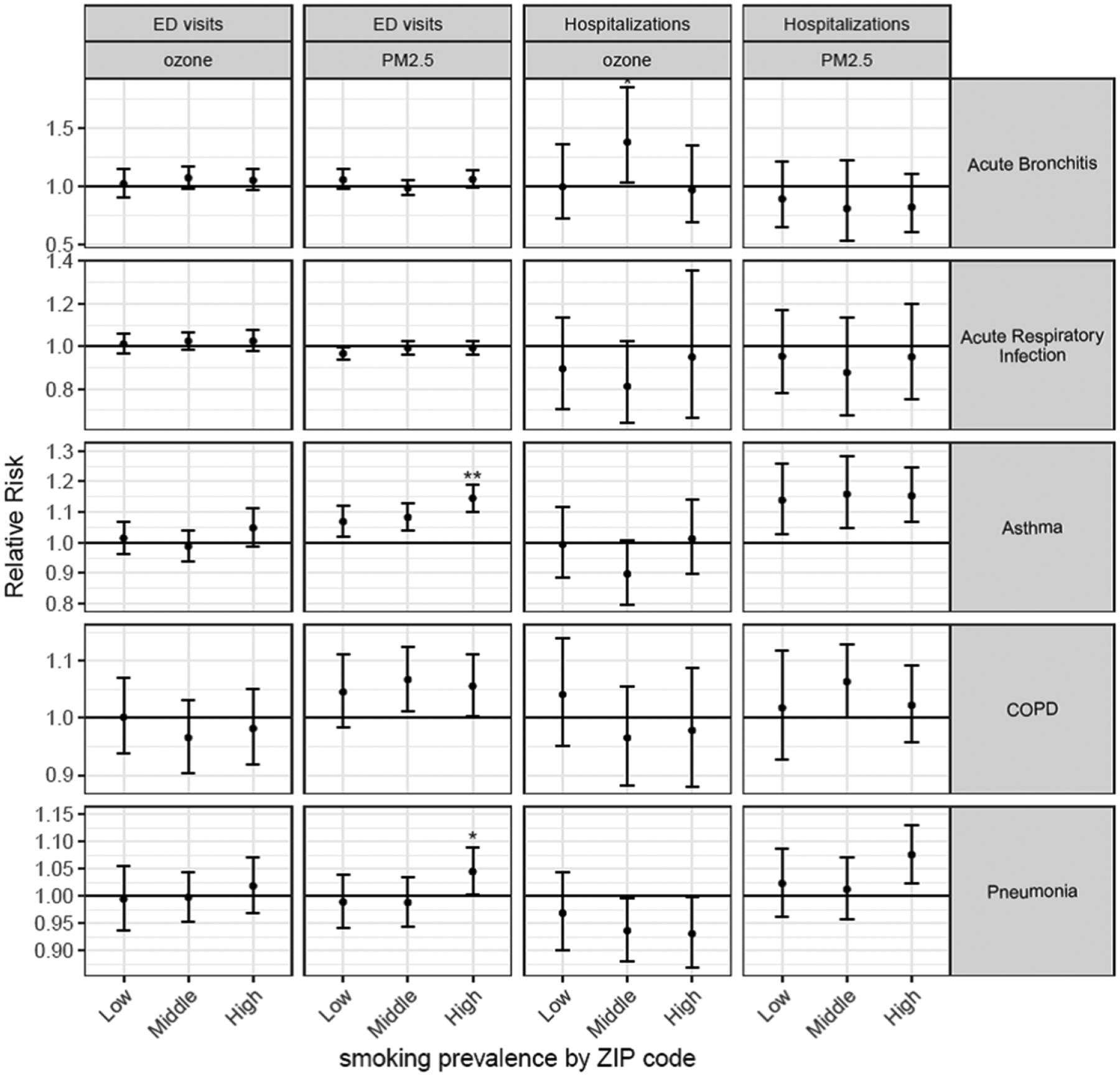
Associations per one *μ*g m^−3^ increase in PM_2.5_ and ozone for ED visits and hospitalizations by levels of ZIP code-level smoking prevalence during the 2008 northern California wildfires. ** = significantly (*p* < 0.05) different from the lowest tertile; * = significantly (*p* < 0.10) different from the lowest tertile.

We observed significant effects of PM_2.5_ during the wildfires on pneumonia hospitalizations and ED visits in ZIP codes with the highest prevalence of smokers whereas the impacts of PM_2.5_ in ZIP codes with lower smoking prevalence were not significant (figure 4). Similar to our other findings, associations between PM_2.5_ during the wildfires and hospitalizations and ED visits for asthma were significant regardless of smoking prevalence level in the ZIP code, but for ED visits the association was significantly higher in the highest smoking tertile compared to the lowest.

Increasing tertiles of some SES measures indicate higher SES (e.g. % expensive homes, % high income) whereas higher values of the other SES measures indicate lower SES.

## 4. Discussion

We observed that asthma ED visits and hospitalizations were associated with PM_2.5_ during the 2008 northern California wildfires in communities of all levels of SES. COPD hospitalizations and ED visits were associated with PM_2.5_ most commonly for ZIP codes with the lowest SES levels. Our finding of significant asthma exacerbations during a wildfire for all ZIP codes, regardless of SES level, adds to the growing understanding of the impact of wildfire smoke on asthma outcomes [45], with some recent studies implying that the dose-response for wildfire smoke may be steeper than for other sources of PM_2.5_ particularly for asthma [46, 47]. Our findings of differential COPD impacts by SES during the 2008 northern California wildfires add to the understanding of differential health risks from wildfire smoke. These findings are increasingly important as wildfire smoke becomes a greater source of air pollution in the western United States [7, 8] and as climate change is increasing the risk of wildfires in this and other global regions [2, 3].

Differential health impacts by SES and race/ethnicity due to air pollution in North America are hypothesized to be due both to higher exposure to air pollution and higher individual susceptibility to air pollution among these communities or some combination thereof [21]. Many studies document higher air pollution exposures in lower SES communities, particularly in North American cities [21, 25], with a few exceptions [21], and in communities of color [48–50]. The historical, economic, and political drivers of these unequal exposures to industrial and vehicular air pollution exposures are complex [51]. In contrast, the spatial patterning of low SES communities and communities of color in relation to wildfire smoke exposure would likely differ among fires and contexts depending on where a fire ignites and the direction of the winds. Our study found differential exposure by SES during this wildfire for some but not all metrics of SES, for example notably higher PM_2.5_ levels in ZIP codes with fewer expensive homes and more low income residents and higher PM_2.5_ in ZIP codes with lower % of residents living in poverty and % unemployed and in ZIP codes with lower levels of the SEP Index and Concentrated Disadvantage [52, 53]. PM_2.5_ concentrations were also higher in ZIP codes with higher numbers of White residents and lower numbers of Black, Hispanic/Latino, and Asian residents. In previous studies examining differential exposure to wildfire smoke, one found higher exposures in counties with larger numbers of elderly Blacks than elderly Whites over five wildfire seasons in the western US [15], another found higher concentrations of PM_2.5_ during wildfires in communities with more White residents [33], whereas another found no relationship between PM_2.5_ from wildfire smoke with county-level median income [54].

We observed higher rates of ED visits for COPD in ZIP codes with lower SES levels, particularly for ZIP codes with higher rates of low-income residents, residents living in poverty, higher rates of unemployed residents, working class residents, and with higher levels of concentrated disadvantage and the SEP index. Not all of the increased health risk in these ZIP codes can be due to increased exposure to PM_2.5_ because we observed higher PM_2.5_ concentrations in ZIP codes with lower levels of poverty, the SEP Index, and Concentrated Disadvantage. The way we conceptualized ‘exposure’ to wildfire smoke in this study used concentrations of total PM_2.5_ at the ZIP code-level during a wildfire event which may not correspond to individual exposures. We did find consistent evidence of effect modification in ER visits for ARI, asthma, and COPD based on the percent in working class jobs. True PM_2.5_ exposures during the wildfire could still be differential by SES due to the ability of people with higher SES to decrease their exposures through use of private rather than public transit, occupations that are primarily indoors compared to outdoors, use of air filtration or air conditioning that filters out air pollution, housing that is less leaky to outdoor air [21], evacuating the area during a wildfire, or other actions that wealth can support. Additionally, the higher associations between COPD ED visits in lower SES ZIP codes could be due more to differences in susceptibility and not just to differential exposure. Increased susceptibility to air pollution among lower SES communities could be due to a combination of higher rates of pre-existing health conditions, psychosocial stress associated with lower SES, material deprivation, lack of health insurance or access to health care, and higher proportions of people in working class jobs.

Very few studies of the health impacts of wildfire smoke exposure have investigated differential impacts by SES or racial composition [11]. A recent meta-analysis found only ten studies from North America that investigated differential health effects of wildfire smoke by population sub-groups [55]. Only three of those investigated effects by SES, and the SES metrics, health outcomes, age groups, and spatial scales differed, making inference across studies challenging. Only two studies to date have investigated differential health impacts of wildfire smoke by race in the United States [14, 15]. Given the growing influence of wildfire smoke on air pollution in the US [7, 8], more research into the differential health impacts of wildfire smoke by SES and race is needed.

Similarly, there are few studies investigating differential acute health impacts by neighborhood-level SES of daily PM_2.5_ exposures on respiratory health outcomes. Much of the literature on differential impacts of air pollution on health due to SES focus either on long-term air pollution exposures or on health outcomes not included in our study. Within short-term studies of differential effects of air pollution on respiratory health by neighborhood-level SES, different findings have been reported with some studies finding higher impacts of air pollution on a health outcome in areas with lower SES, higher SES, or some studies finding no difference in effects by SES [56]. There are many methodological choices in all of these studies that could explain these differences in findings such as the scale of neighborhood used (e.g. county, ZIP code, census block), the SES metrics used, the cut-points used to look at different levels of SES (e.g. quartiles, dichotomous by median value) [56], the air pollutant analyzed [56, 57], how the air pollutant was measured (e.g. nearest monitor, modeled) [58], and/or by the health outcome analyzed (both diagnosis code and severity level such as hospitalization, ED visit, etc) [57].

To our knowledge, our study is the first to analyze the impacts of wildfire smoke exposure by area-level smoking prevalence. We found higher risk of ED visits for asthma and pneumonia and hospitalizations for pneumonia associated with increases in PM_2.5_ during the wildfires in the ZIP codes with the highest smoking prevalence. Previous studies have investigated whether PM_2.5_, not specifically from wildfires, is differentially associated with respiratory health based on whether someone is a smoker or not, with mixed evidence. Some studies document that never smokers as compared to current or former smokers experienced declines in lung function associated with PM_2.5_ [59] and were more likely to have symptoms of chronic bronchitis associated with PM_2.5_ [60]. Other studies, however, have found no significant interaction by smoking status on the association between respiratory hospitalizations and air pollution [61], or that there was a stronger association between PM_2.5_ and prevalence of asthma among smokers compared to non-smokers [62]. It is not clear if the differences in findings across studies are due to the different respiratory health outcomes investigated, methodological differences, or differences in underlying health or air pollution context of the population studied, as these all differed across these studies. Regardless of those differences, all studies that we know of investigating differential impact of PM_2.5_ on respiratory health by smoking status all used data at the individual-level, which was not available for our study. We had to assume that area-level smoking prevalence implies that more of the people in ZIP codes with higher smoking prevalence were exposed to either first-hand or second-hand cigarette smoke, however we have no evidence that those who were hospitalized or visited the ED for asthma or pneumonia were indeed smokers, and we would not want to imply that the finding in our study at the ZIP code-level applies at the individual level. Further research on susceptibility to wildfire smoke, and air pollution in general, by exposure to first-hand and second-hand cigarette smoke is warranted.

Although many studies of wildfire smoke and health now calculate wildfire specific PM_2.5_ separately from non-wildfire derived PM_2.5_ in their models, we did not do that, instead relying on total PM_2.5_ during the wildfire period to assess the influence of the wildfire air pollution on health. Although some studies have documented differential impacts of wildfire-derived PM_2.5_ on health outcomes compared to non-wildfire PM_2.5_ [46, 47]—and this could be important to explore further especially as wildfires are increasingly burning more human-made materials in addition to vegetation which could make the chemistry of the PM_2.5_ more toxic [63]—we argue that people, and their health, are impacted by the total PM_2.5_ (and other pollutants) that they breathe, and not each component separately, such that we think that assessing the impact of air pollution during a wildfire to be important. Indeed, given that wildfire smoke is an increasing amount of the PM_2.5_ that people are breathing in the western US [7], one way to protect people’s health from this growing threat would be to strengthen regulation to decrease other sources of PM_2.5_.

While our study adds to the scant literature on differential respiratory health impacts during wildfire events, it has some limitations. We focused on only one major wildfire event in one region. Although the wildfires investigated here were significant at the time, wildfires in 2017, 2018, and 2020 in California and the Pacific Northwest caused PM_2.5_ levels much higher than those observed in this study. Increasingly studies are investigating multiple wildfires in many locations over longer periods of time [64–66] to increase statistical power. We suggest that those studies also investigate differential impacts by SES, race/ethnicity, and smoking to determine if our findings hold in other contexts. We were also constrained by aggregate data and therefore could not investigate differential impacts by individual-level rather than area-level characteristics. Finally, our analysis may raise concerns about multiple testing due to the number of outcomes and effect modifiers considered. We employed an inclusive approach and did not adjust for multiple comparisons to explore patterns in the data. To avoid spurious conclusions, we highlighted consistent findings across multiple effect modifiers for a given health outcome rather than focus on any one finding of statistical significance.

Despite these limitations, our study makes some important contributions. Our study investigated a variety of measures of SES and more racial/ethnic groups than in previous studies. We also investigated the role of O_3_ during a wildfire, which few studies have investigated from a health perspective [67]. The lack of significant associations between O_3_ and respiratory health outcomes by SES/racial composition levels found here should not be taken as definitive that O_3_ during a wildfire does not affect respiratory health; different fires can affect ground-level O_3_ in different ways [68] depending on fire characteristics, plume heights, distance away from the fire, background concentrations of precursors of ozone production and many other characteristics [69]. We also used machine-learning-based metrics of exposure assessment for both pollutants, thus allowing spatiotemporal concentration estimates. Finally, we included acute bronchitis and acute respiratory infections, two respiratory health endpoints that have not been sufficiently studied in the wildfire-health literature [11].

## 5. Conclusion

We found associations between PM_2.5_ during this wildfire and asthma hospitalizations and ED visits regardless of the ZIP code SES level, but increased risk of ED visits for COPD associated with PM_2.5_ during the wildfire in lower SES ZIP codes across multiple SES measures. This was found despite evidence that PM_2.5_ concentrations during the wildfires were not uniformly higher in lower SES ZIP codes during this specific wildfire. We infer from this that at least some of the differential health impact by SES from the wildfires is due to differential susceptibility that SES confers on one’s health, although noting that actual PM_2.5_ exposures, which differ from the outside PM_2.5_ concentrations that we used, may differ by SES. SES may impact respiratory health susceptibility through material deprivation, psychosocial stress, access to health care or ability to manage one’s health conditions. We also found higher risk for exacerbations of asthma and pneumonia associated with wildfire PM_2.5_ in ZIP codes with the highest smoking prevalence, which could imply that being exposed to more cigarette smoke may predispose people to be more affected by wildfire smoke. More research is needed on the differential impacts of wildfire smoke exposure on health to better target public health interventions during increasingly common and devastating wildfire events as the climate changes.

## Supplementary Material

Reid_2023_ERH_Supplement

## Figures and Tables

**Table 1.** Mean and range of daily rates (per 1000 people) of hospitalization and ED visit by ZIP code in study period.

	Hospitalizations			ED visits		
Respiratory outcomes	Mean	Minimum	Maximum	Mean	Minimum	Maximum
Asthma	0.002	0	10.989	0.014	0	71.429
COPD	0.003	0	3.067	0.007	0	17.857
Pneumonia	0.007	0	23.810	0.016	0	71.429
Acute bronchitis	0	0	1.372	0.007	0	62.5
Acute respiratory infections	0	0	0.515	0.028	0	71.429

**Table 2.** Median and range of SES and smoking prevalence by ZIP code across the study area.

	Median	Minimum	Maximum
% Expensive homes	6.3	0	100
% Low income	18.3	0	100
% High income	3.6	0	52.0
% Poverty	10.5	0	100
SEP Index^a^	−0.6	−5.9	11.9
Concentrated Disadvantage^a^	−0.2	−1	5.9
% Unemployed	6.1	0	100
% White	71.1	9.6	98.6
% Black	1.1	0	60.2
% Asian	2.9	0	64.1
% Hispanic/Latino	13.4	0.9	99.3
% Working class	85.4	40	100
Smoking prevalence	17.8	5.3	44.2

a The SEP Index and the Concentrated Disadvantage Index are based on summing *Z*-scores. They range from negative values (less disadvantage) to positive values (more disadvantage).

**Table 3.** Associations between each SES or race/ethnicity variable and PM_2.5_ or ozone from univariate linear regressions.

	PM_2.5_			Ozone		
Characteristic	Beta	95% CI^a^	*p*-value	Beta	95% CI^a^	*p*-value
% Expensive homes	−0.070	−0.083, −0.056	<0.001	−0.00033	−0.00034, −0.00031	<0.001
% Low income	0.044	0.031, 0.058	<0.001	0.00028	0.00027, 0.00029	<0.001
% High income	−0.168	−0.200, *−*0.135	<0.001	−0.00078	−0.00081, −0.00076	<0.001
% Poverty	−0.028	−0.045, *−*0.012	<0.001	0.00027	0.00026, 0.00028	<0.001
SEP Index	−0.285	−0.351, *−*0.220	<0.001	0.00158	0.00153, 0.00164	<0.001
Concentrated Disadvantage	−1.164	−1.430, *−*0.899	<0.001	0.00253	0.00230, 0.00276	<0.001
% Unemployed	−0.024	−0.045, *−*0.002	0.031	0.00041	0.00039, 0.00043	<0.001
% White	0.184	0.175, 0.192	<0.001	0.00019	0.00018, 0.00020	<0.001
% Black	−0.191	−0.216, *−*0.166	<0.001	−0.00051	−0.00053, −0.00049	<0.001
% Asian	−0.157	−0.173, *−*0.140	<0.001	−0.00055	−0.00056, −0.00054	<0.001
% Hispanic/Latino	−0.171	−0.179, *−*0.163	<0.001	0.00001	0.00001, 0.00002	<0.001
% Working class	0.079	0.058, 0.099	<0.001	0.00068	0.00067, 0.00070	<0.001
Smoking prevalence	70.656	66.436, 74.875	<0.001	0.08732	0.08364, 0.09099	<0.001

a CI = confidence interval

## Data Availability

The data cannot be made publicly available upon publication because they contain sensitive personal information. The data that support the findings of this study are available upon reasonable request from the authors.
