# Supplementary material for: Effect modification of the association between fine particulate air pollution during a wildfire event and respiratory health by area-level measures of socio-economic status, race/ethnicity, and smoking prevalence
Source: Environ Res Health. Author manuscript; Available in PMC 2024 Feb 8. (PMC10852067; doi:10.1088/2752-5309/acc4e1)
Supplement: Reid_2023_ERH_Supplement [file NIHMS1963165-supplement-Reid_2023_ERH_Supplement.pdf]

Supplementary Material for: "Effect modification of the association between fine particulate air pollution during a wildfire event and respiratory health by area-level measures of socio-economic status, race/ethnicity, and smoking prevalence"

Table of Contents

Supplementary Text 1. Air Pollution Exposure Model Information 2
Supplementary Text 2. Calculation of SES Metrics 2
Supplementary Text 3. Sensitivity Analysis into the Categorization of SES Variables..... 2
Supplementary Figure 1. Associations between PM2.5 and hospitalizations by levels of ZIP code-level race/ethnicity during the 2008 northern California wildfires.................... 3
Supplementary Figure 2. Associations between $PM_{2.5}$ and ED visits by levels of ZIP code-level race/ethnicity during the 2008 northern California wildfires........................... 4
Supplementary Figure 3. Associations between ozone and hospitalizations by levels of ZIP code-level SES during the 2008 northern California wildfires.................................. 5
Supplementary Figure 5. Associations between ozone and hospitalizations by levels of ZIP code-level race/ethnicity during the 2008 northern California wildfires.................... 7
Supplementary Figure 6. Associations between ozone and ED visits by levels of ZIP code-level race/ethnicity during the 2008 northern California wildfires........................... 8
Supplementary Table 1. Associations per one µg/m^3 increase in $PM_{2.5}$ and hospitalizations by levels of ZIP code-level SES and race/ethnicity during the 2008 northern California wildfires... 1
Supplementary Table 2. Associations per one µg/m^3 increase in $PM_{2.5}$ and ED visits by levels of ZIP code-level SES and race/ethnicity during the 2008 northern California wildfires. 4

Supplementary Text 1. Air Pollution Exposure Model Information

Environmental covariates used in the air pollution prediction models included static land use information and daily satellite air pollution measures, meteorological data, and chemical transport model output. The dependent variable in each model was daily observations from ground monitors for the pollutant of interest. After performing 10-fold cross-validation (CV) on ten commonly used machine learning algorithms, it was determined that the generalized boosting model (GBM) fit both pollutants the best. The GBM CV-R^2 was 0.78 and 0.73 for $PM_{2.5}$ and O_3 respectively. More methodological details can be found in the original publications (Reid et al., 2015; Watson et al., 2019).

Supplementary Text 2. Calculation of SES Metrics

We obtained estimates by ZIP code of total population, percent of the population over 65, percent unemployed, percent of the population below the poverty line, percent of the population on welfare, median household income, percent of the population living in owner-occupied housing, house values for all owner-occupied housing, percent of female householders, and the percent of the population in each racial/ethnic group (percentages of White, Black, Hispanic/Latino, Asian, American Indian and Alaska Native, Native Hawaiian and Other Pacific Islander) from the 2000 U.S. Census. We calculated the working class percent of the population by subtracting percentages of the population in white collar occupations (Census categories "Finance, insurance, real estate and rental and leasing" and "Professional, scientific, management, administrative, and waste management services") from 100%. This is derived from previous work by (Krieger et al., 2002).

We used ZIP code-level median income, percent of the population over 65, percent of the population under age 5, and percent of the population that was non-white as confounders that could address differential population risk for respiratory and cardiovascular diseases at the ZIP code-level. We used the other data to calculate area-level measures of socio-economic status and racial and ethnic composition as described below.

The SEP Index is calculated by summing z-scores for the following measures of SES: non-professional vocation + unemployment rate + below poverty + less than HS education + homes worth >= $750,000 (this last term is reverse coded before summing) (Krieger et al., 2002). Higher values of the SEP Index denote lower SES. Concentrated Disadvantage is calculated by summing z-scores of unemployment rate, percent living below poverty, percent of female-headed households, percent on welfare, and percent black (Graif & Sampson, 2009).

Supplementary Text 3. Sensitivity Analysis into the Categorization of SES Variables

When analyzing the distributions of the variables, we found that some of them were skewed such that using quantiles (categories with the same number of observations), in our case tertiles, could have some categories that had a large range compared to others that had a small range. Using equal intervals creates the same range within each category but can yield fewer observations in some categories than in others, which can yield unstable results. As none of our distributions showed clear clusters through visual inspection, we opted to use tertiles (categories with equal numbers of observations), leading to more stable results across categories. We used k-means clustering to define SES categories as a sensitivity analysis.

The results of our sensitivity analyses that used k-means clustering rather than tertiles to create the groups for the effect modification analysis by SES and race/ethnicity are in the Supplemental

Material (Supplemental Figures 6-13). Although the specific relative risks changed based on the clustering method, the overall pattern of findings remained the same, thus bolstering our confidence in the findings by tertile. Regardless of level of SES, across almost all SES metrics, asthma hospitalization and ED visits were associated with $PM_{2.5}$ during the fires. With the k-means clustering, the highest SES group was often non-significantly associated with $PM_{2.5}$, but the confidence intervals were wider due to smaller numbers of ZIP codes in the higher group (ranged from 1-7% of all ZIP codes) for each SES variable (which does not necessarily mean the most socially disadvantaged group) except for working class in which the lowest k-means group had the smallest percentage of ZIP codes (15% of all ZIP codes) and thus the largest confidence interval. Similarly, for the SES variables, the category representing the lowest SES category showed the strongest association between COPD ED visits with $PM_{2.5}$ during the wildfire period.

Supplementary Figure 1. Associations between PM2.5 and hospitalizations by levels of ZIP code-level race/ethnicity during the 2008 northern California wildfires.

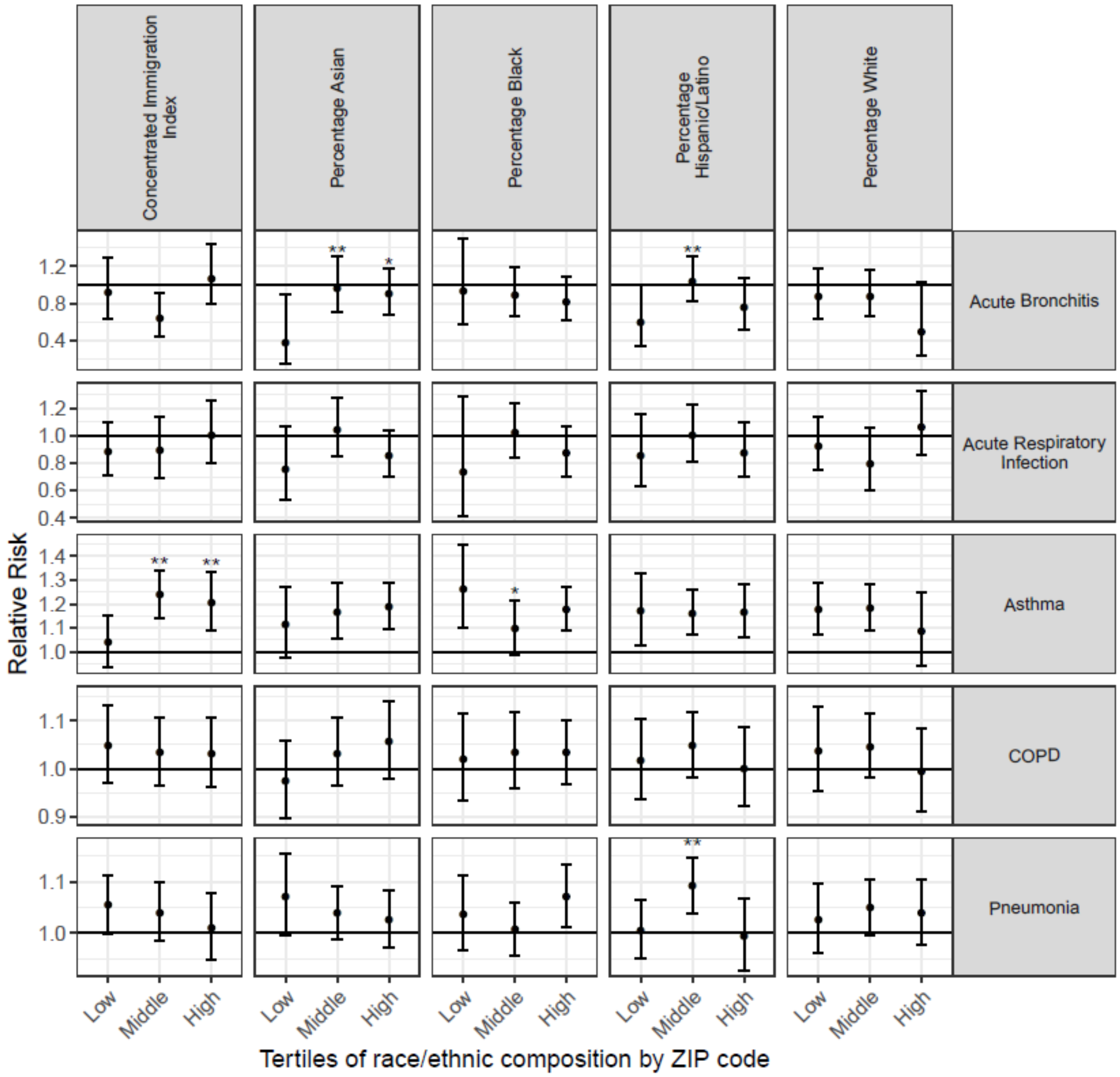

** = significantly ($p < 0.05$) different from the lowest tertile;
* = significantly ($p < 0.10$) different from the lowest tertile

Supplementary Figure 2. Associations between $PM_{2.5}$ and ED visits by levels of ZIP code-level race/ethnicity during the 2008 northern California wildfires.

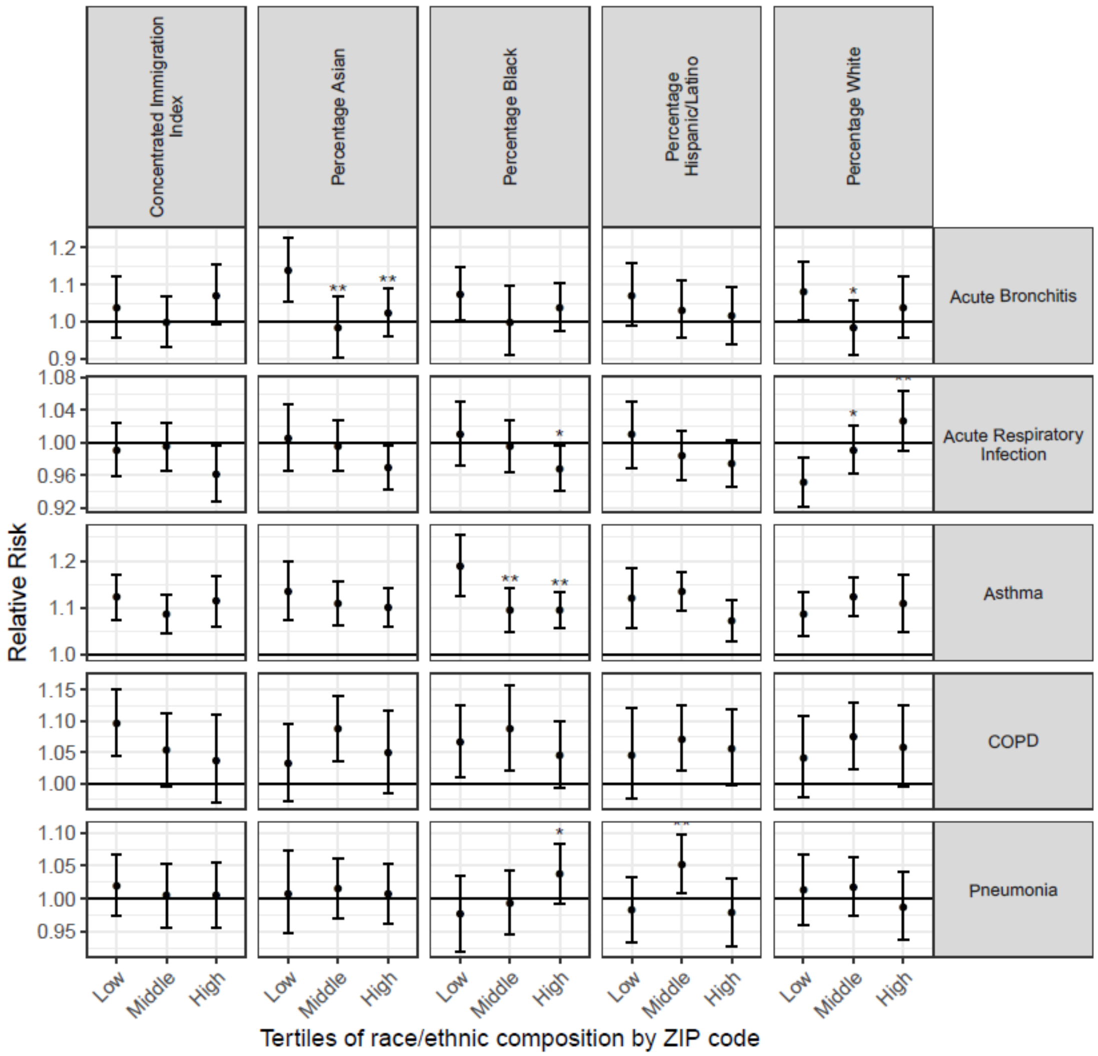

** = significantly (p < 0.05) different from the lowest tertile;
* = significantly (p < 0.10) different from the lowest tertile

Supplementary Figure 3. Associations between ozone and hospitalizations by levels of ZIP code-level SES during the 2008 northern California wildfires.

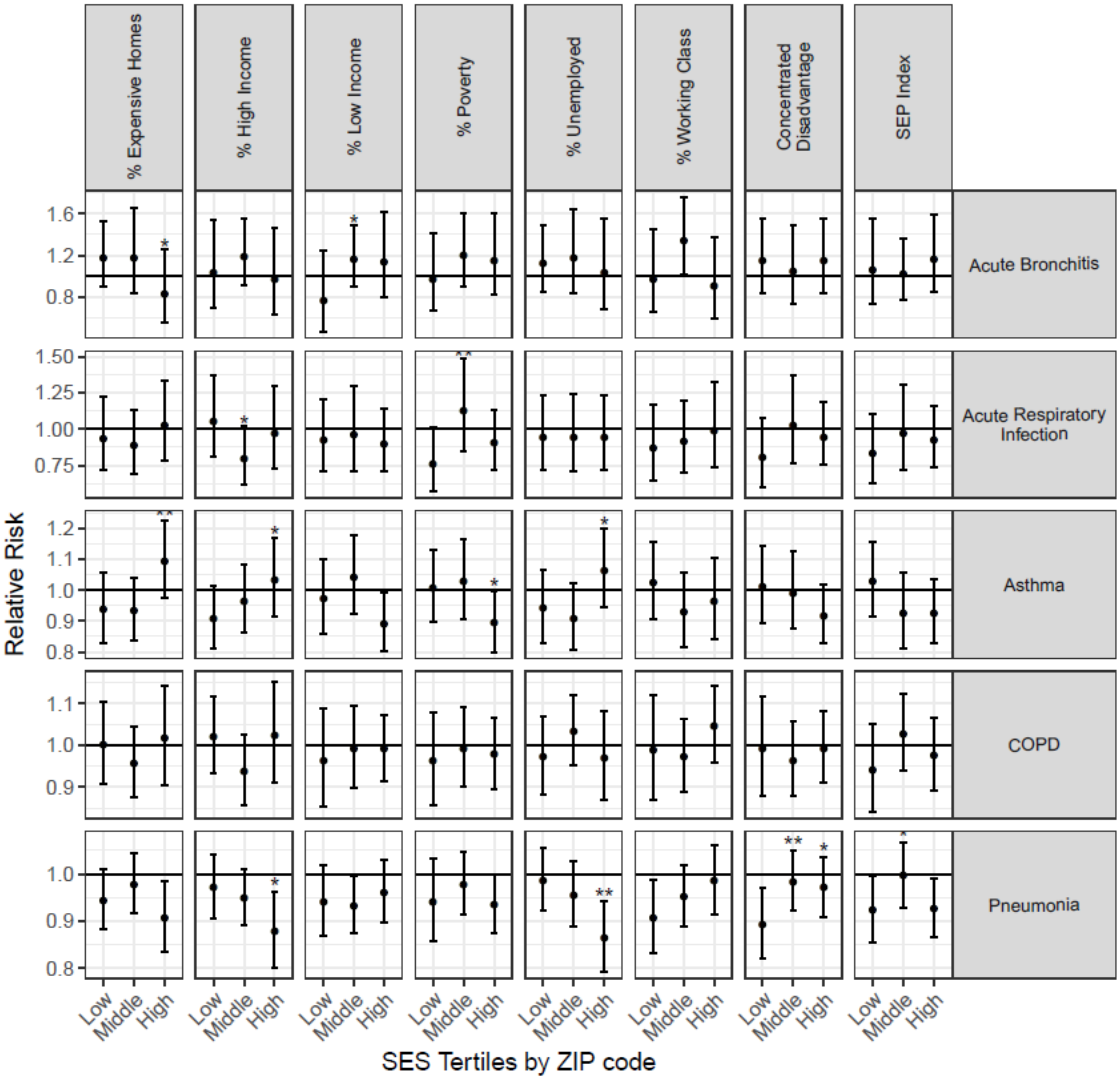

** = significantly (p < 0.05) different from the lowest tertile;
* = significantly (p < 0.10) different from the lowest tertile

Supplementary Figure S4. Associations between Ozone and ED visits by levels of ZIP code-level SES during the 2008 northern California wildfires

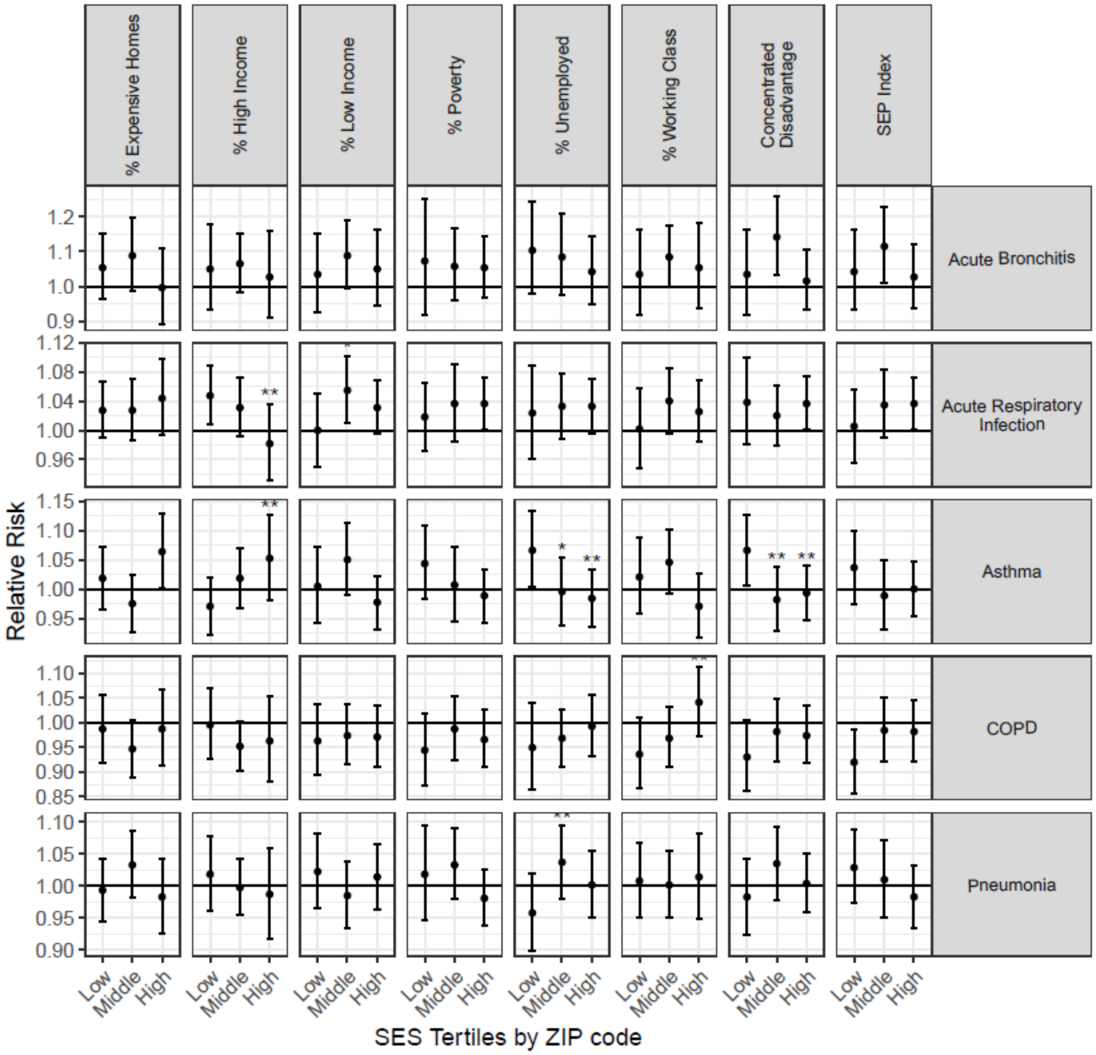

** = significantly (p < 0.05) different from the lowest tertile;
* = significantly (p < 0.10) different from the lowest tertile

Supplementary Figure 5. Associations between ozone and hospitalizations by levels of ZIP code-level race/ethnicity during the 2008 northern California wildfires.

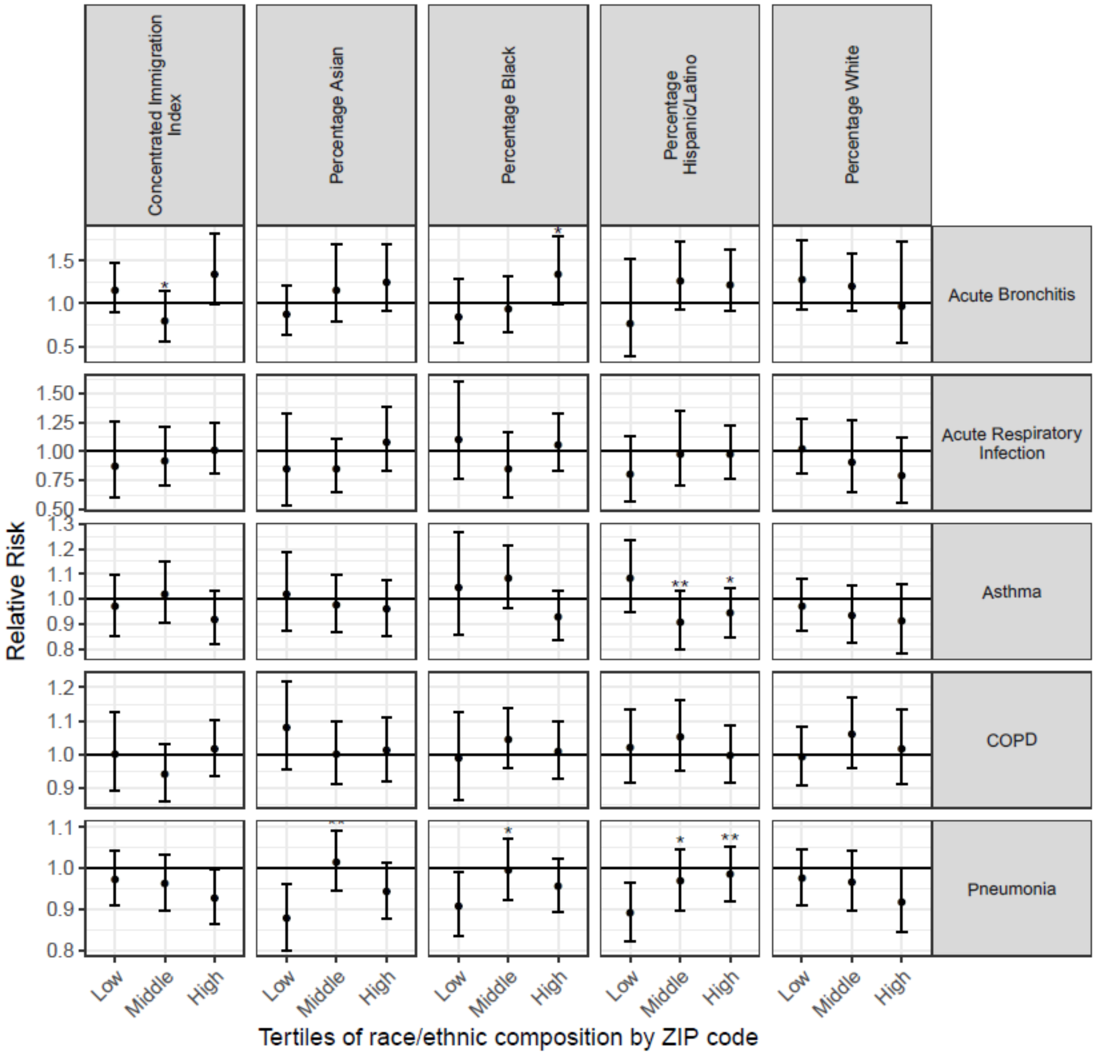

** = significantly (p < 0.05) different from the lowest tertile;
* = significantly (p < 0.10) different from the lowest tertile

Supplementary Figure 6. Associations between ozone and ED visits by levels of ZIP code-level race/ethnicity during the 2008 northern California wildfires.

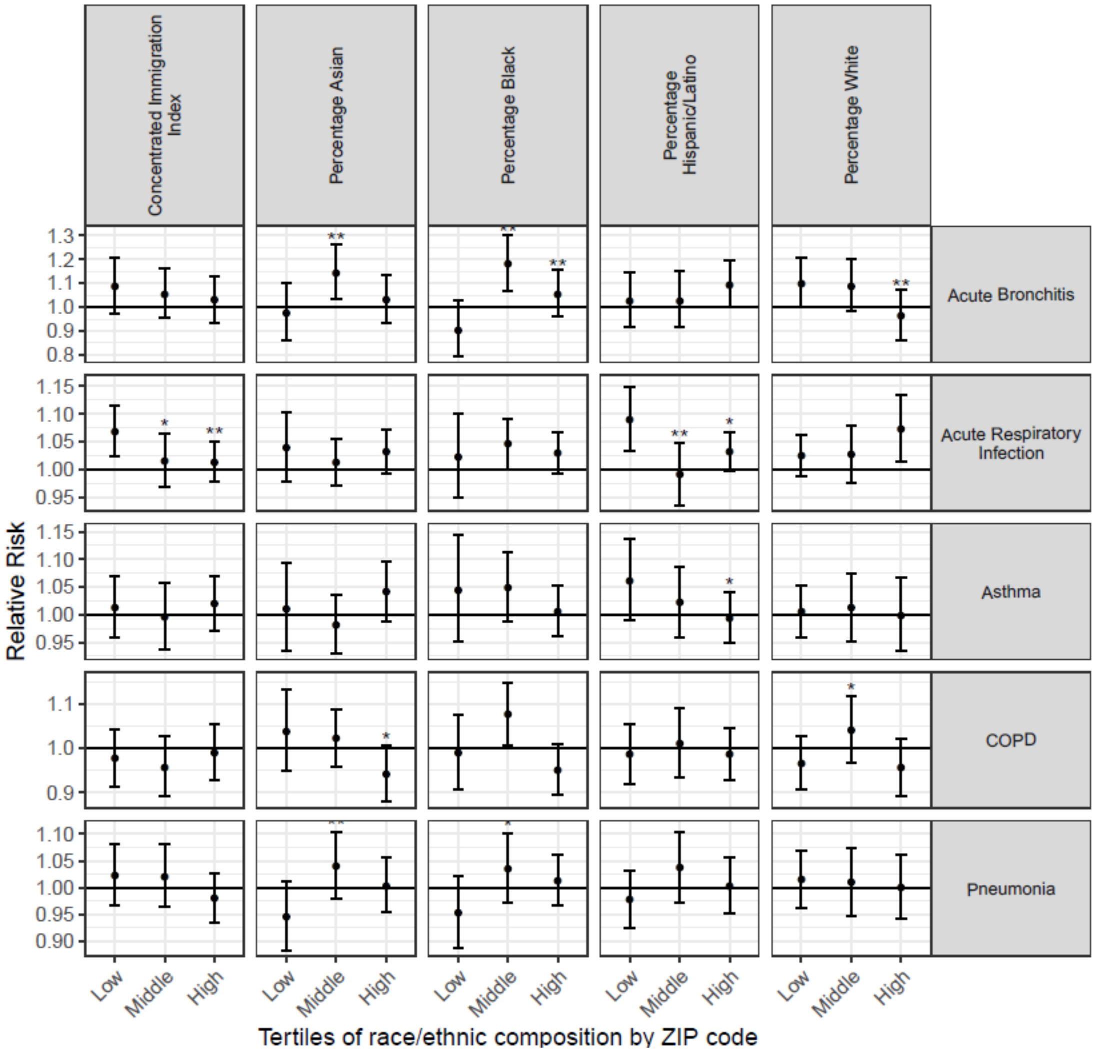

** = significantly ($p < 0.05$) different from the lowest tertile;
* = significantly ($p < 0.10$) different from the lowest tertile

Supplementary Table 1. Associations per one μg/m^3 increase in $PM_{2.5}$ and hospitalizations by levels of ZIP code-level SES and race/ethnicity during the 2008 northern California wildfires.

		RR (95% CI)				
		Lowest Tertile	Middle Tertile	Highest Tertile	p-value 1	p-value 2
acute bronchitis	% Expensive Homes	0.88 (0.62, 1.24)	0.84 (0.59, 1.19)	0.86 (0.63, 1.16)	0.84	0.9
	% High Income	0.77 (0.48, 1.26)	0.84 (0.60, 1.16)	0.94 (0.72, 1.22)	0.79	0.48
	% Low Income	0.86 (0.64, 1.17)	0.82 (0.60, 1.13)	0.94 (0.65, 1.35)	0.83	0.73
	% poverty	0.98 (0.74, 1.31)	0.63 (0.43, 0.93)	0.98 (0.71, 1.35)	0.07	0.98
	% unemployment	0.88 (0.63, 1.21)	0.83 (0.57, 1.21)	0.89 (0.66, 1.20)	0.83	0.93
	% working class	1.00 (0.76, 1.31)	0.89 (0.66, 1.2)	0.67 (0.41, 1.10)	0.57	0.17
	SEP Index	0.87 (0.63, 1.19)	0.80 (0.56, 1.15)	0.93 (0.67, 1.29)	0.74	0.74
	Concentrated Disadvantage	0.89 (0.63, 1.25)	0.81 (0.60, 1.10)	0.86 (0.60, 1.24)	0.68	0.91
	% Asian	*0.37 (0.15, 0.90)*	0.97 (0.71, 1.31)	0.90 (0.69, 1.18)	0.04	0.06
	% Black	0.93 (0.58, 1.49)	0.89 (0.66, 1.20)	0.82 (0.62, 1.09)	0.87	0.64
	% White	0.87 (0.64, 1.18)	0.87 (0.66, 1.16)	0.49 (0.24, 1.03)	0.98	0.16
	% Hispanic/Latino	0.59 (0.35, 1.00)	1.04 (0.82, 1.32)	0.75 (0.52, 1.08)	0.05	0.45
	Smoking Prevalence	0.89 (0.65, 1.22)	0.81 (0.53, 1.22)	0.82 (0.61, 1.10)	0.71	0.69
ARI	% Expensive Homes	*0.68 (0.50, 0.94)*	1.03 (0.85, 1.24)	0.97 (0.77, 1.21)	0.02	0.07
	% High Income	1.03 (0.85, 1.26)	0.78 (0.59, 1.03)	0.94 (0.76, 1.16)	0.09	0.5
	% Low Income	0.94 (0.76, 1.16)	0.84 (0.63, 1.12)	0.97 (0.80, 1.18)	0.53	0.79
	% poverty	0.93 (0.77, 1.12)	0.96 (0.73, 1.27)	0.91 (0.72, 1.14)	0.83	0.88
	% unemployment	1.01 (0.82, 1.25)	0.81 (0.60, 1.10)	0.92 (0.74, 1.15)	0.21	0.5
	% working class	0.91 (0.72, 1.15)	0.81 (0.64, 1.02)	1.09 (0.90, 1.33)	0.45	0.21
	SEP Index	0.95 (0.77, 1.16)	0.95 (0.75, 1.21)	0.90 (0.70, 1.15)	0.96	0.73
	Concentrated Disadvantage	0.90 (0.71, 1.13)	0.97 (0.77, 1.22)	0.90 (0.72, 1.13)	0.61	0.99
	% Asian	0.75 (0.53, 1.07)	1.04 (0.85, 1.27)	0.85 (0.70, 1.03)	0.1	0.52

	% Black	0.73 (0.42, 1.29)	1.02 (0.84, 1.24)	0.87 (0.70, 1.07)	0.26	0.57
	% White	0.92 (0.75, 1.13)	0.79 (0.60, 1.05)	1.06 (0.86, 1.32)	0.37	0.31
	% Hispanic/Latino	0.85 (0.63, 1.16)	1.00 (0.81, 1.23)	0.88 (0.70, 1.09)	0.37	0.88
	Smoking Prevalence	0.95 (0.78, 1.17)	0.88 (0.68, 1.14)	0.95 (0.75, 1.20)	0.59	0.98
Asthma	% Expensive Homes	**1.15 (1.03, 1.29)**	**1.15 (1.07, 1.25)**	**1.17 (1.07, 1.28)**	0.95	0.78
	% High Income	**1.18 (1.06, 1.3)**	**1.16 (1.07, 1.26)**	**1.15 (1.05, 1.26)**	0.83	0.71
	% Low Income	**1.14 (1.05, 1.24)**	**1.17 (1.06, 1.29)**	**1.18 (1.07, 1.29)**	0.71	0.63
	% poverty	**1.12 (1.03, 1.21)**	**1.22 (1.10, 1.34)**	**1.16 (1.05, 1.28)**	0.13	0.49
	% unemployment	**1.19 (1.09, 1.29)**	**1.15 (1.03, 1.28)**	**1.17 (1.07, 1.29)**	0.6	0.86
	% working class	**1.15 (1.05, 1.26)**	**1.21 (1.11, 1.31)**	1.10 (0.98, 1.23)	0.42	0.51
	SEP Index	**1.16 (1.06, 1.26)**	**1.18 (1.08, 1.3)**	**1.16 (1.05, 1.28)**	0.68	1
	Concentrated Disadvantage	**1.12 (1.02, 1.24)**	**1.20 (1.10, 1.31)**	**1.16 (1.06, 1.26)**	0.28	0.63
	% Asian	1.11 (0.97, 1.27)	**1.17 (1.05, 1.29)**	**1.19 (1.09, 1.29)**	0.57	0.4
	% Black	**1.26 (1.10, 1.45)**	1.09 (0.99, 1.21)	**1.18 (1.09, 1.27)**	0.09	0.35
	% White	**1.18 (1.07, 1.29)**	**1.18 (1.09, 1.28)**	1.08 (0.94, 1.25)	0.95	0.33
	% Hispanic/Latino	**1.17 (1.03, 1.33)**	**1.16 (1.07, 1.26)**	**1.16 (1.06, 1.28)**	0.92	0.96
	Smoking Prevalence	**1.14 (1.03, 1.26)**	**1.16 (1.05, 1.28)**	**1.15 (1.07, 1.25)**	0.80	0.84
COPD	% Expensive Homes	1.05 (0.98, 1.12)	1.01 (0.93, 1.08)	1.08 (1.00, 1.17)	0.38	0.58
	% High Income	1.02 (0.95, 1.09)	1.06 (0.99, 1.14)	1.04 (0.96, 1.14)	0.36	0.61
	% Low Income	1.02 (0.93, 1.10)	1.03 (0.96, 1.11)	1.06 (1.00, 1.13)	0.76	0.36
	% poverty	1.01 (0.93, 1.09)	1.01 (0.93, 1.10)	**1.09 (1.03, 1.15)**	0.95	0.12
	% unemployment	1.01 (0.94, 1.08)	1.07 (1.00, 1.14)	1.02 (0.93, 1.11)	0.21	0.92
	% working class	0.99 (0.91, 1.09)	1.03 (0.96, 1.10)	1.06 (1.00, 1.14)	0.55	0.22
	SEP Index	1.02 (0.94, 1.11)	1.01 (0.94, 1.09)	**1.09 (1.02, 1.15)**	0.82	0.21
	Concentrated Disadvantage	1.03 (0.94, 1.14)	0.99 (0.93, 1.07)	**1.09 (1.03, 1.15)**	0.48	0.36
	% Asian	0.97 (0.90, 1.06)	1.03 (0.96, 1.11)	1.06 (0.98, 1.14)	0.28	0.15

	% Black	1.02 (0.93, 1.11)	1.03 (0.96, 1.12)	1.03 (0.97, 1.10)	0.81	0.83
	% White	1.04 (0.95, 1.13)	1.05 (0.98, 1.11)	0.99 (0.91, 1.08)	0.87	0.46
	% Hispanic/Latino	1.02 (0.94, 1.10)	1.05 (0.98, 1.12)	1.00 (0.92, 1.09)	0.54	0.79
	Smoking Prevalence	1.02 (0.93, 1.12)	1.06 (1.00, 1.13)	1.02 (0.96, 1.09)	0.41	0.93
pneumonia	% Expensive Homes	1.04 (0.98, 1.11)	1.05 (1.00, 1.11)	1.02 (0.96, 1.08)	0.8	0.54
	% High Income	**1.08 (1.02, 1.14)**	1.03 (0.98, 1.09)	1.01 (0.95, 1.07)	0.25	0.09
	% Low Income	1.03 (0.98, 1.10)	0.98 (0.92, 1.05)	**1.09 (1.04, 1.15)**	0.2	0.13
	% poverty	1.03 (0.97, 1.09)	1.01 (0.95, 1.08)	**1.07 (1.02, 1.13)**	0.7	0.26
	% unemployment	1.02 (0.97, 1.08)	**1.11 (1.06, 1.16)**	1.00 (0.94, 1.06)	0.02	0.51
	% working class	1.04 (0.98, 1.10)	1.00 (0.95, 1.06)	**1.10 (1.04, 1.16)**	0.4	0.13
	SEP Index	1.04 (0.98, 1.10)	1.03 (0.98, 1.09)	1.05 (1.00, 1.11)	0.94	0.66
	Concentrated Disadvantage	1.01 (0.94, 1.07)	1.03 (0.97, 1.09)	**1.07 (1.02, 1.13)**	0.62	0.09
	% Asian	1.07 (0.99, 1.15)	1.04 (0.99, 1.09)	1.03 (0.97, 1.08)	0.48	0.32
	% Black	1.04 (0.97, 1.11)	1.01 (0.96, 1.06)	**1.07 (1.01, 1.13)**	0.48	0.45
	% White	1.03 (0.96, 1.10)	1.05 (1.00, 1.10)	1.04 (0.98, 1.10)	0.56	0.78
	% Hispanic/Latino	1.01 (0.95, 1.06)	**1.09 (1.04, 1.15)**	0.99 (0.93, 1.07)	0.02	0.79
	Smoking Prevalence	1.02 (0.96, 1.09)	1.01 (0.96, 1.07)	**1.08 (1.02, 1.13)**	0.78	0.18

Bolded Values are significant positive associations at the p<0.05 level between PM2.5 and the outcome for that level of effect modifier. Italicized Values are significant negative associations at the p<0.05 level between PM2.5 and the outcome for that level of effect modifier.
P-value1 = p-value for interaction term comparing the middle tertile to the lowest tertile of the effect modifier
P-value2 = p-value for interaction term comparing highest tertile to lowest tertile of the effect modifier

Supplementary Table 2. Associations per one µg/m^3 increase in $PM_{2.5}$ and ED visits by levels of ZIP code-level SES and race/ethnicity during the 2008 northern California wildfires.

		RR (95% CI)				
		Lowest Tertile	Middle Tertile	Highest Tertile	p-value1	p-value2
acute bronchitis	% Expensive Homes	1.01 (0.94, 1.09)	1.04 (0.97, 1.12)	1.05 (0.97, 1.14)	0.61	0.51
	% low income	1.02 (0.95, 1.09)	0.98 (0.92, 1.05)	**1.10 (1.01, 1.20)**	0.47	0.11
	% high income	1.03 (0.96, 1.11)	1.03 (0.96, 1.11)	1.04 (0.97, 1.13)	0.95	0.82
	% poverty	1.03 (0.95, 1.11)	0.93 (0.87, 1.01)	**1.14 (1.06, 1.22)**	0.06	0.04
	SEP Index	0.99 (0.92, 1.06)	1.03 (0.94, 1.12)	**1.10 (1.02, 1.19)**	0.4	0.02
	Concentrated Disadvantage	1.04 (0.96, 1.12)	0.98 (0.91, 1.05)	**1.09 (1.01, 1.18)**	0.23	0.3
	% unemployment	1.03 (0.95, 1.11)	0.99 (0.91, 1.07)	**1.09 (1.01, 1.17)**	0.46	0.26
	% working class	1.01 (0.94, 1.09)	1.04 (0.97, 1.12)	1.04 (0.96, 1.12)	0.54	0.64
	% Black	1.07 (1.00, 1.15)	1.00 (0.91, 1.10)	1.04 (0.97, 1.10)	0.19	0.43
	% Asian	**1.14 (1.05, 1.23)**	0.98 (0.90, 1.07)	1.02 (0.96, 1.09)	0.01	0.03
	% White	1.08 (1.00, 1.16)	0.98 (0.91, 1.06)	1.04 (0.96, 1.12)	0.05	0.4
	% Hispanic/Latino	1.07 (0.99, 1.16)	1.03 (0.96, 1.11)	1.01 (0.94, 1.09)	0.46	0.29
	Smoking Prevalence	1.05 (0.97, 1.15)	0.98 (0.92, 1.04)	1.06 (0.98, 1.14)	0.14	0.95
acute respiratory infection	% Expensive Homes	0.99 (0.96, 1.03)	0.97 (0.95, 1.00)	0.98 (0.95, 1.01)	0.37	0.55
	% low income	0.97 (0.94, 1.00)	0.98 (0.95, 1.01)	1.00 (0.97, 1.04)	0.64	0.12
	% high income	1.02 (0.99, 1.06)	0.98 (0.95, 1.00)	0.96 (0.93, 0.99)	0.04	0
	% poverty	0.97 (0.94, 1.00)	0.99 (0.96, 1.03)	0.98 (0.95, 1.02)	0.22	0.54
	SEP Index	*0.97 (0.94, 0.99)*	0.99 (0.96, 1.02)	1.00 (0.96, 1.04)	0.2	0.11
	Concentrated Disadvantage	0.97 (0.94, 1.00)	0.99 (0.96, 1.02)	0.99 (0.95, 1.02)	0.35	0.45
	% unemployment	*0.96 (0.93, 0.99)*	0.99 (0.96, 1.02)	1.01 (0.98, 1.05)	0.15	0.02
	% working class	*0.96 (0.93, 0.99)*	0.98 (0.96, 1.01)	1.03 (1.00, 1.07)	0.19	0
	% Black	1.01 (0.97, 1.05)	1.00 (0.96, 1.03)	0.97 (0.94, 1.00)	0.53	0.07
	% Asian	1.01 (0.97, 1.05)	1.00 (0.97, 1.03)	0.97 (0.94, 1.00)	0.69	0.13
	% White	*0.95 (0.92, 0.98)*	0.99 (0.96, 1.02)	1.03 (0.99, 1.06)	0.05	0
	% Hispanic/Latino	1.01 (0.97, 1.05)	0.98 (0.95, 1.01)	0.97 (0.95, 1.00)	0.31	0.14

	Smoking Prevalence	0.97 (0.94, 1.00)	0.99 (0.96, 1.03)	0.99 (0.96, 1.02)	0.21	0.20
asthma	% Expensive Homes	**1.13 (1.09, 1.18)**	**1.09 (1.05, 1.13)**	**1.10 (1.05, 1.15)**	0.12	0.23
	% low income	**1.07 (1.02, 1.12)**	**1.15 (1.11, 1.2)**	**1.11 (1.06, 1.15)**	0.01	0.21
	% high income	**1.14 (1.1, 1.18)**	**1.10 (1.05, 1.14)**	**1.08 (1.04, 1.13)**	0.14	0.07
	% poverty	**1.09 (1.05, 1.14)**	**1.14 (1.09, 1.19)**	**1.09 (1.05, 1.13)**	0.19	0.93
	SEP Index	**1.10 (1.06, 1.14)**	**1.12 (1.07, 1.17)**	**1.10 (1.06, 1.16)**	0.55	0.88
	Concentrated Disadvantage	**1.09 (1.04, 1.14)**	**1.14 (1.09, 1.19)**	**1.10 (1.06, 1.14)**	0.17	0.87
	% unemployment	**1.09 (1.05, 1.14)**	**1.12 (1.07, 1.16)**	**1.12 (1.07, 1.17)**	0.51	0.47
	% working class	**1.06 (1.02, 1.11)**	**1.12 (1.08, 1.17)**	**1.14 (1.09, 1.2)**	0.03	0.01
	% Black	**1.19 (1.12, 1.25)**	**1.09 (1.05, 1.14)**	**1.09 (1.06, 1.13)**	0.02	0.01
	% Asian	**1.14 (1.07, 1.2)**	**1.11 (1.06, 1.16)**	**1.10 (1.06, 1.14)**	0.48	0.33
	% White	**1.09 (1.04, 1.13)**	**1.12 (1.08, 1.17)**	**1.11 (1.05, 1.17)**	0.2	0.56
	% Hispanic/Latino	**1.12 (1.06, 1.18)**	**1.13 (1.09, 1.18)**	**1.07 (1.03, 1.12)**	0.7	0.2
	Smoking Prevalence	**1.07 (1.02, 1.12)**	**1.08(1.04, 1.13)**	**1.15 (1.10, 1.19)**	0.66	0.02
COPD	% Expensive Homes	**1.08 (1.03, 1.13)**	1.05 (0.99, 1.11)	**1.08 (1.01, 1.15)**	0.35	0.97
	% low income	1.01 (0.95, 1.07)	**1.06 (1.01, 1.12)**	**1.11 (1.06, 1.17)**	0.2	0.01
	% high income	**1.08 (1.03, 1.14)**	**1.08 (1.03, 1.14)**	1.02 (0.95, 1.09)	0.95	0.12
	% poverty	1.00 (0.94, 1.06)	1.06 (1.00, 1.12)	**1.12 (1.06, 1.17)**	0.14	0
	SEP Index	1.02 (0.97, 1.07)	1.06 (1.00, 1.13)	**1.12 (1.06, 1.17)**	0.26	0.01
	Concentrated Disadvantage	1.02 (0.96, 1.09)	1.05 (1.00, 1.11)	**1.10 (1.05, 1.16)**	0.45	0.05
	% unemployment	1.04 (0.98, 1.10)	1.04 (0.98, 1.11)	**1.10 (1.05, 1.16)**	0.82	0.07
	% working class	1.00 (0.94, 1.06)	**1.06 (1.01, 1.12)**	**1.11 (1.05, 1.17)**	0.09	0.01
	% Black	**1.07 (1.01, 1.13)**	**1.09 (1.02, 1.16)**	1.05 (0.99, 1.10)	0.64	0.58
	% Asian	1.03 (0.97, 1.10)	**1.09 (1.04, 1.14)**	1.05 (0.98, 1.12)	0.16	0.71
	% White	1.04 (0.98, 1.11)	**1.07 (1.02, 1.13)**	1.06 (1.00, 1.13)	0.39	0.69
	% Hispanic/Latino	1.05 (0.98, 1.12)	**1.07 (1.02, 1.12)**	1.06 (1.00, 1.12)	0.56	0.81
	Smoking Prevalence	1.05 (0.98, 1.11)	**1.07(1.01, 1.12)**	**1.06 (1.00, 1.11)**	0.59	0.79
pn eu	% Expensive Homes	1.02 (0.98, 1.07)	1.03 (0.99, 1.07)	0.97 (0.92, 1.02)	0.83	0.12

% low income	1.03 (0.98, 1.08)	0.96 (0.91, 1.00)	1.05 (1.00, 1.09)	0.02	0.55
% high income	1.05 (1.00, 1.10)	1.00 (0.96, 1.04)	0.98 (0.94, 1.03)	0.09	0.04
% poverty	1.00 (0.95, 1.05)	1.00 (0.95, 1.05)	1.03 (0.99, 1.08)	0.97	0.27
SEP Index	1.02 (0.97, 1.07)	0.99 (0.94, 1.04)	1.02 (0.98, 1.07)	0.31	0.92
Concentrated Disadvantage	0.98 (0.93, 1.03)	1.01 (0.97, 1.06)	1.04 (0.99, 1.08)	0.3	0.07
% unemployment	0.97 (0.92, 1.02)	1.02 (0.97, 1.07)	1.05 (1.00, 1.09)	0.15	0.02
% working class	1.00 (0.96, 1.05)	0.99 (0.95, 1.04)	1.04 (1.00, 1.09)	0.79	0.21
% Black	0.98 (0.92, 1.04)	0.99 (0.95, 1.04)	1.04 (0.99, 1.08)	0.63	0.09
% Asian	1.01 (0.95, 1.07)	1.01 (0.97, 1.06)	1.01 (0.96, 1.05)	0.85	0.97
% White	1.01 (0.96, 1.07)	1.02 (0.97, 1.06)	0.99 (0.94, 1.04)	0.88	0.48
% Hispanic/Latino	0.98 (0.93, 1.03)	**1.05 (1.01, 1.10)**	0.98 (0.93, 1.03)	0.03	0.9
Smoking Prevalence	0.99 (0.94, 1.04)	0.99 (0.94, 1.04)	1.05 (1.00, 1.09)	0.98	0.08

Bolded Values are significant positive associations at the p<0.05 level between PM2.5 and the outcome for that level of effect modifier. Italicized Values are significant negative associations at the p<0.05 level between PM2.5 and the outcome for that level of effect modifier.
P-value1 = p-value for interaction term comparing the middle tertile to the lowest tertile of the effect modifier
P-value2 = p-value for interaction term comparing highest tertile to lowest tertile of the effect modifier
